# Prediction of Human Disease Genes by Human-Mouse Conserved Coexpression Analysis

**DOI:** 10.1371/journal.pcbi.1000043

**Published:** 2008-03-28

**Authors:** Ugo Ala, Rosario Michael Piro, Elena Grassi, Christian Damasco, Lorenzo Silengo, Martin Oti, Paolo Provero, Ferdinando Di Cunto

**Affiliations:** 1 Molecular Biotechnology Center, Department of Genetics, Biology and Biochemistry, University of Turin, Turin, Italy; 2 Department of Human Genetics and Centre for Molecular and Biomolecular Informatics, University Medical Centre Nijmegen, Nijmegen, The Netherlands; Lilly Singapore Centre for Drug Discovery, Singapore

## Abstract

**Background:**

Even in the post-genomic era, the identification of candidate genes within loci associated with human genetic diseases is a very demanding task, because the critical region may typically contain hundreds of positional candidates. Since genes implicated in similar phenotypes tend to share very similar expression profiles, high throughput gene expression data may represent a very important resource to identify the best candidates for sequencing. However, so far, gene coexpression has not been used very successfully to prioritize positional candidates.

**Methodology/Principal Findings:**

We show that it is possible to reliably identify disease-relevant relationships among genes from massive microarray datasets by concentrating only on genes sharing similar expression profiles in both human and mouse. Moreover, we show systematically that the integration of human-mouse conserved coexpression with a phenotype similarity map allows the efficient identification of disease genes in large genomic regions. Finally, using this approach on 850 OMIM loci characterized by an unknown molecular basis, we propose high-probability candidates for 81 genetic diseases.

**Conclusion:**

Our results demonstrate that conserved coexpression, even at the human-mouse phylogenetic distance, represents a very strong criterion to predict disease-relevant relationships among human genes.

## Author Summary

One of the most limiting aspects of biological research in the post-genomic era is the capability to integrate massive datasets on gene structure and function for producing useful biological knowledge. In this report we have applied an integrative approach to address the problem of identifying likely candidate genes within loci associated with human genetic diseases. Despite the recent progress in sequencing technologies, approaching this problem from an experimental perspective still represents a very demanding task, because the critical region may typically contain hundreds of positional candidates. We found that by concentrating only on genes sharing similar expression profiles in both human and mouse, massive microarray datasets can be used to reliably identify disease-relevant relationships among genes. Moreover, we found that integrating the coexpression criterion with systematic phenome analysis allows efficient identification of disease genes in large genomic regions. Using this approach on 850 OMIM loci characterized by unknown molecular basis, we propose high-probability candidates for 81 genetic diseases.

## Introduction

In the last two decades, positional cloning has been remarkably successful in the identification of genes involved in human disorders. More recently, our ability to map genetic disease loci has strikingly increased due to the availability of the entire genome sequence. Nevertheless, once a disease locus has been mapped, the identification of the mutation responsible for the phenotype still represents a very demanding task, because the mapped region may typically contain hundreds of candidates [1]. Accordingly, many phenotypes mapped on the genome by linkage analysis are not yet associated to any validated disease gene (850 OMIM entries for phenotypes with unknown molecular basis had at least one associated disease locus on July 2^nd^, 2007). Therefore, the definition of strategies that can pinpoint the most likely targets to be sequenced in patients is of critical importance [1]. Many different strategies have been proposed to prioritize genes located in critical map intervals. Some of the methods so far developed rely on the observation that disease genes tend to share common global properties, which can be deduced directly by absolute and comparative sequence analysis [2]. However, most of the available prioritization strategies are based on the widely accepted idea that genes and proteins of living organisms deploy their functions as part of sophisticated functional modules, based on a complex series of physical, metabolic and regulatory interactions [3],[4]. Although this principle has been extensively used even in the pre-genome era to identify the critical players of many different biological phenomena, the present availability of genome-scale information on gene function, protein-protein interactions and gene expression in different experimental models allows unprecedented opportunities for approaching the prioritization problem with greater efficiency.

In theory, the use of functional gene annotations would represent the most straightforward approach for candidate prioritization. However, although this strategy may be very useful in selected cases [5],[6], at the present stage it has clear limitations, either because it overlooks non-annotated genes [6],[7] or because it is not evident how the annotated functions of the candidates relate to the disease phenotype. Therefore, computational methods less biased toward already consolidated knowledge, may have strong advantages [1].

In particular, protein-protein interaction maps and gene coexpression data from microarray experiments represent extremely rich sources of potentially relevant information.

Recently, the direct integration of a very heterogeneous human interactome with a text mining-based map of phenotype similarity has allowed the prediction of high confidence candidates within large disease-associated loci [8].

Although this approach is highly efficient, it is clearly not exhaustive because very close functional relationships between genes and proteins are possible in the absence of direct molecular binding. In addition, the protein-protein interaction space is currently under-sampled and many genuine biological interactions have not yet been identified in experiments. Conversely, high-throughput experiments are known to result in a large fraction of false positives. The consistently low overlap of protein-protein interactions between large-scale experiments, even when the same proteins are considered, is testament to these problems [9]. Finally, many of the known protein-protein interactions have been ascertained through low-throughput experiments and are thus strongly biased towards better-studied proteins [10].

Since genes involved in the same functions tend to show very similar expression profiles, coexpression analysis could be a very powerful approach for inferring functional relationships, which may correlate with similar disease phenotypes. Accordingly, global analyses have shown that genes highly coexpressed across microarray experiments display very similar functional annotation [11]. However, with notable exceptions [12], so far the coexpression criterion has not been employed very successfully for the prediction of genetic disease candidates and has been used to this purpose only in combination with other independent evidence [5],[13].

The noisy nature of high-throughput gene expression datasets may represent one of the possible explanations for this shortcoming. Moreover, even when the coexpression of two genes is reproducibly observed under a high number of experimental conditions, this does not necessarily imply that the genes are functionally related. For instance, extensive meta analysis of microarray data across different species has revealed that neighboring genes are more likely to be coexpressed than genes encoded in distant genomic regions, even if they are not functionally related in any obvious manner [14],[15].

Phylogenetic conservation has been previously proposed as a very strong criterion to identify functionally relevant coexpression links among genes [16],[17]. Indeed, significant coexpression of two or more orthologous genes is very likely due to selective advantage, strongly suggesting a functional relation. Therefore, conserved coexpression could be a much stronger criterion than single species coexpression to relate genes involved in similar disease phenotypes.

In this report we show that conserved coexpression and phenome analysis can be effectively integrated to produce accurate predictions of human disease genes. Using this approach we were able to select a small number of strong candidates for 81 human diseases, corresponding to a wide spectrum of different phenotypes.

## Materials and Methods

**Figure 1:**
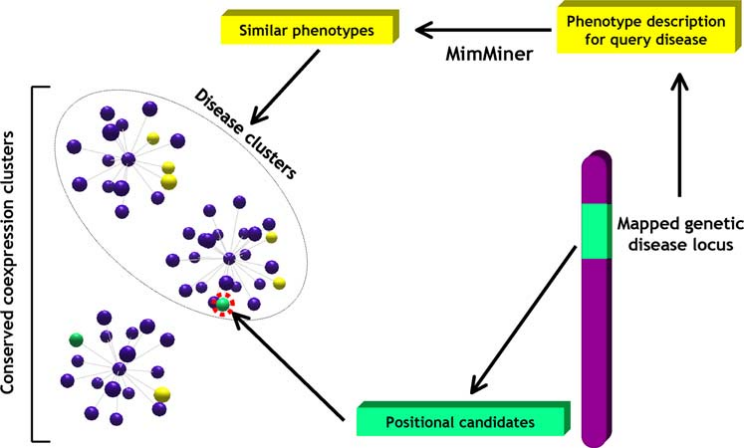
Identification of candidate disease genes by means of conserved co-expression clusters. A locus with positional candidates (green bar) associated with a disease of unknown molecular basis (yellow) is screened for one or more candidate genes (green sphere) that appear in a conserved coexpression cluster (purple spheres) together with at least two other genes known to be involved in similar phenotypes (yellow spheres), as defined by the MimMiner similarity score.

Figure 1 schematically illustrates our approach.

### Generation of Conserved Coexpression Networks

We have generated two human-mouse conserved coexpression networks (CCN), based on cDNA and oligonucleotide microarray platforms, respectively.

In the first case, the starting data were log-transformed values of human and mouse ratiometric experiments, downloaded from the Stanford Microarray Database (SMD) [18] (4129 experiments for 102296 EST probes for human and 467 experiments for 80595 EST probes for mouse). The resulting network will be referred to as ‘Stanford’ in the following (Text S1).

In the second case (‘Affy’, Text S2), the network was based on previously described series of normal tissue Affymetrix microarray experiments from human [19] (353 experiments corresponding to 65 different tissues for 46241 probe-sets associated to a known gene) and mouse [20] (122 experiments corresponding to 61 tissues for 19692 probe-sets). In the human case, the Affymetrix experiments corresponding to the same tissue were averaged to compensate for the different number of replicates available for the various tissues.

In both cases, we used the same procedure to generate a final CCN. In particular, we first generated single species gene coexpression networks (SCN) and then integrated them on the basis of human-mouse orthology, as detailed below.

SCNs were generated by first calculating the Pearson correlation coefficients of every row in the expression matrix (cDNA probe or Affymetrix probe-set) with all other rows. A directed edge was established from row r_1_ to row r_2_ if r_2_ fell within the top 1% rows in terms of correlation with r_1_. The threshold was first chosen on the basis of a previous study, showing that such 1% interval is most significantly enriched in terms of functionally relevant coexpression [16]. Moreover, we confirmed that using a more stringent 0.5% threshold results in strongly reduced sensitivity (data not shown).

These directed networks where then converted into undirected SCNs by mapping the rows to the corresponding Entrez Gene identifiers [21]: an edge is established between two Entrez gene IDs G_1_ and G_2_ if there is at least one edge from a row assigned to G_1_ to a row assigned to G_2_ and vice versa. The correspondence between probe-sets and Entrez gene IDs for the SMD data was established using Unigene (build 190 and 152 for human and mouse, respectively). For Affymetrix data we used the annotation files provided by Affymetrix for each platform.

Finally, CCNs were built from SCNs by mapping every Entrez Gene identifier to the corresponding Homologene cluster (build 55) and retaining only the cases in which a one-to-one correspondence could be established between the human and mouse Entrez gene IDs appearing in the SCNs.

### Phenotype Correlation

Genetic disease phenotypes described in OMIM where correlated on the basis of MimMiner [22]. MimMiner assigns a similarity score to all pairs of OMIM phenotype records, based on the text mining analysis of their phenotype descriptions [22]. Two phenotypes were defined to be similar if their score was at least 0.4, because biologically meaningful relationships were mostly detected in phenotype pairs with a similarity score equal or greater than this value [22]. About 1% of all possible pairs of phenotypes included in MimMiner pass this threshold.

### Global Properties of the CCNs

**Figure 2:**
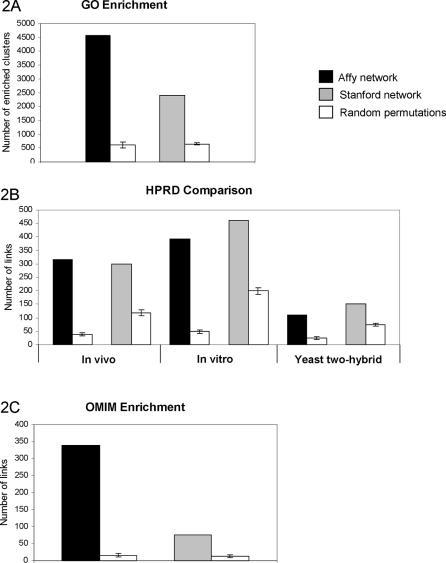
Comparison of the Affy and Stanford networks with functional, physical interaction, and disease-related information. (A) Prevalence of functionally related coexpression clusters (see Materials and Methods). (B) Number of edges of the CCN joining proteins previously shown to physically interact by different techniques, as deduced by the HPRD database. (C) Number of edges of the indicated networks connecting genes involved in Mendelian phenotypes sharing a MimMiner score of 0.4 or higher. In each case, the results for the actual CCNs are compared to the results averaged on 100 randomized CCNs, with error bars representing the standard deviation of the latter.

The analysis of the CCNs was based on the construction of coexpression clusters, defined as a given gene (the center of the cluster) plus its nearest neighbors in the conserved coexpression network, thus obtaining one cluster for each gene. The prevalence of genes joining functionally related genes in the CCNs was tested by analyzing the prevalence of Gene Ontology terms within coexpression clusters, compared to the same prevalence in randomized coexpression clusters. We counted the number of coexpression clusters for which at least one Gene Ontology term was significantly overrepresented (P-value less than 10^−4^ with exact Fisher test), and compared this number with the same number averaged over 100 randomized CCNs. This was done separately for the Affy and Stanford networks, and the results are shown in Figure 2A.

The overlap between CCNs and protein interaction networks was evaluated by downloading the list of known interactions between human proteins from HPRD [23],[24]. To take into account the different experimental methods on which the HPRD interactions are based and their varying degree of reliability, we analyzed separately in-vivo, in vitro and yeast double hybrid interactions. In each case, separately for the Affy and Stanford networks, we compared the overlap between the CCNs and the protein interaction network to the same overlap averaged over 100 randomized CCNs. The results are shown in Figure 2B.

Finally to verify whether the CCNs were enriched in edges joining genes causing similar phenotypes, we constructed a network of human genes in which an edge was placed between every pair of genes known to be involved in the same disease or in diseases with MimMiner similarity score at least equal to 0.4. The mapping between OMIM phenotypes and genes known to cause them was obtained from Ensembl, version 45 [25]. We then evaluated the overlap between this network and the CCNs, again compared to the same overlap averaged over 100 randomized CCNs (Figure 2C).

### Identification of Candidate Genes in Disease Loci

The lists of genes contained in OMIM loci of unknown molecular basis were obtained from Ensembl, version 45 [25]. To identify likely candidates for a given disease-associated genomic locus, we first extracted from the networks disease-relevant conserved coexpression clusters. A cluster was considered relevant to a given disease *d* if it contained at least two genes experimentally known to cause phenotypes similar to *d.* These clusters will be called ‘disease clusters’ in the following. The genes of the disease clusters, which are also contained in the map interval of the locus associated to *d*, were retained for scoring as described below.

### Scoring and Estimation of the False Discovery Rate

The size of the coexpression clusters and of the loci are very heterogeneous: coexpression clusters contain between 2 and 186 genes while loci associated to OMIM diseases with unknown molecular basis vary between 3 and 2153 genes. Therefore the genes selected in the previous step were assigned a probabilistic score based on the null hypothesis in which the coexpression clusters are random sets of genes. The score is essentially the probability that the two events leading to the identification of a candidate gene for a disease occur by chance. The two events are (1) the presence in a coexpression cluster of at least 2 genes known to be involved in phenotypes similar to the disease in question and (2) the presence in the same coexpression cluster of a gene located within the locus relevant to the disease. First, we computed a P-value *p_1_* for associating a cluster to the given disease by chance. This P-value is given by the cumulative hypergeometric distribution considering: the number *R_DC_* of genes linked to similar phenotypes that have been found in the cluster (at least 2 to associate the cluster with the disease); the number *R_all_* of genes in the network that are linked to similar phenotypes; the number *G_DC_* of genes in the cluster; and the total number of genes *G_all_* in the network:
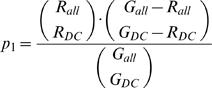
Second, we computed the P-value *p_2_* of the overlap between the disease cluster and the genetic locus associated to the disease. This P-value is given by the cumulative hypergeometric distribution considering: the number *L_DC_* of genes in the locus that are also present in the given disease cluster; the number *L_all_* of genes in the locus that are present in the network; and *G_DC_* and *G_all_* are defined as above:
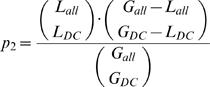
In the null hypothesis, associating a cluster with a disease and finding a gene in the cluster that belongs to the appropriate orphan locus are independent events. Thus, the total score for a predicted candidate is given by the product p_1_ • p_2_. When a candidate is found in more than one disease cluster, we consider only the lowest (best) score.

The cutoff on such scores was determined by estimating the false discovery rate (FDR) for each possible cutoff using 100 randomized CCNs per dataset: The false discovery rate is defined as the ratio between the average number of predictions made using randomized CCNs and the number of predictions made using the real CCN, with the same cutoff. The cutoffs thus obtained for a 10% FDR were 4.49·10^−6^ for the Affy and 2.67·10^−6^ for the Stanford network, respectively.

### Precision and Leave-One-Out Procedure

To estimate the precision of our procedure we used a leave-one-out strategy: For every gene experimentally associated to an OMIM phenotype we constructed artificial loci centered on the disease gene, and removed all associations between this particular phenotype and all the genes known to cause it, so as to simulate a phenotype with unknown molecular basis. The association between phenotypes similar to the one under examination and the corresponding genes was instead retained.

In order to take into account the variability of locus sizes we constructed artificial loci of various sizes, by taking the disease gene plus the N closest genes on each side of the chromosome (according to their start position on the chromosome). The artificial loci thus contained up to 2N+1 genes, but could contain fewer genes when the disease gene was close to one of the chromosome ends. In the following discussion, such artificial loci will be denoted as N20, N50, … N500 for locus sizes up to 41, 101 … 1001 genes, respectively. This range of locus sizes was chosen based on the observed size distribution of orphan loci: OMIM loci for diseases with unknown molecular basis contain an average number of about 273 genes (median: 180 genes).

We considered the disease gene as correctly identified if it was selected as a candidate by our method with the same score threshold that we used for the orphan loci. The precision is defined as the ratio between the number of cases with correctly identified disease genes and the number of cases with at least one selected candidate, that is, the fraction of cases with selected candidates in which the disease gene was among the candidate list.

## Results

### Generation and Global Properties of Two Different Human-Mouse Conserved Coexpression Networks

Conserved coexpression has been previously reported to be an efficient criterion to identify functionally related genes [16],[17]. Therefore, to discover new relationships between human genes with a high potential relevance for disease phenotypes, we produced the two human-mouse gene coexpression networks described above, covering different platforms and experimental conditions. In particular, the Stanford network (supporting file S1) was generated from data based on cDNA platforms, corresponding mostly to experiments performed on tumor cell lines. In contrast, the Affy network (Text S2) was derived from normal tissue data, generated on Affymetrix platforms in two independent studies [19],[20]. The Stanford network has 8512 nodes (genes) and 56397 edges, with an average connectivity of 13.2 edges per node. The Affy network is composed of 12766 nodes and 155403 edges, with an average connectivity of 24.3 edges per node. Both networks contain a large connected component of 2305 and 4122 genes, respectively, with some other small connected components containing only a few nodes.

As expected from previous studies on gene coexpression networks [26], the two networks are topologically similar to other biological networks, characterized by the existence of a few highly connected nodes (hubs), but they show a connectivity distribution more similar to an exponential law than to a scale-free one (data not shown). More importantly, if compared with 100 random permutations, both networks show a strong prevalence of edges between genes that are annotated to the same Gene Ontology (GO) keyword (Figure 2A). This confirms that human-mouse conserved coexpression is a valuable criterion to identify functionally related genes. Accordingly, both networks show a highly significant overlap with protein-protein interactions reported in the Human Protein Reference Database (HPRD) [23],[24] (Figure 2B).

Since many genes, such as those involved in basic cellular functions, should be coexpressed regardless of the particular experimental situation, we would expect the two networks to have many common links. Indeed, they share 2305 edges, between the 7332 common nodes, which represents a striking overlap (the randomized Affy networks had on average 88.4 edges in common with the Stanford network, with a standard deviation of 8.7). On the other hand, the large number of specific links that characterize the two networks indicates that they provide highly complementary information.

Finally, to evaluate the capability of conserved coexpression to link genes involved in similar disease phenotypes, we measured in both networks the prevalence of links between genes associated to phenotypes with similar descriptions [22]. Interestingly, both networks showed a strong enrichment, if compared with the average number obtained from the randomized networks (Figure 2C). We concluded that the two networks represent complementary resources that could efficiently predict disease-relevant relationships among human genes.

### Integration of Coexpression and Phenotypic Information to Identify Likely Candidate Genes in Disease Loci

The high prevalence of links between genes involved in similar disease phenotypes, observed in both networks, suggests that they could provide valuable information to identify likely candidates in mapped disease loci. Therefore, we devised an algorithm that integrates our CCNs with phenotype and mapping information to predict candidate disease genes in large genomic regions (Figure 1). As detailed in Materials and Methods, the procedure is based on the extraction from the network of the disease clusters, which we consider to be associated to a given disease since they contain at least two genes involved in similar phenotypes. The genes that are present in both the OMIM phenotype loci and the corresponding disease clusters are considered as candidates and assigned a score based on the size of the locus and of the disease cluster. Randomized runs allowed us to select a score threshold corresponding to a 10% FDR.

**Figure 3:**
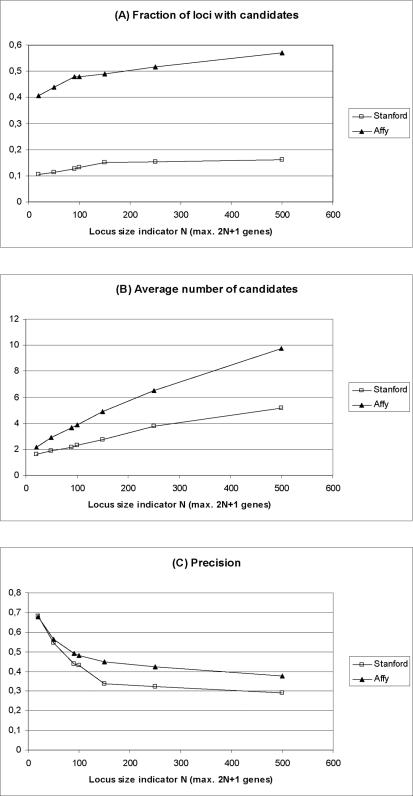
Performance of the identification of candidate disease genes as determined by a leave-one-out strategy. Artificial loci of different sizes were constructed around known disease genes as explained in the text. (A) Fraction of the artificial loci for which it was possible to identify at least one candidate gene, as a function of the locus size. (B) Average number of candidates in the loci for which at least one candidate was identified, as a function of the locus size. (C) Precision as a function of the locus size. Precision is determined as the ratio between the number of loci whose candidate list contained the starting disease gene and the number of loci with candidates. Filled triangles indicate the results obtained with the Affy network, while empty boxes refer to the Stanford network.

To evaluate how our procedure could perform on the loci characterized by unknown molecular basis, we applied a leave-one-out strategy to all the Ensembl genes associated to at least one OMIM disease ID, by constructing artificial loci of variable size around each gene. We then measured the fraction of artificial loci for which we obtained at least one candidate (Figure 3A), the average number of candidates found for these loci (Figure 3B) and the precision (Figure 3C), defined as explained in Materials and Methods. Of the 1762 disease genes contained in OMIM, we could analyze 1426, whose associated phenotypes are present in the MimMiner similarity matrix.

The precision obtained obviously decreases when the size of the artificial loci is increased. However, it is interesting to notice that, while for the smallest artificial loci the precision was excellent (about 68% for both networks), even with the largest artificial loci it was still remarkable (37.5% for the Affy and 29.1% for the Stanford networks, respectively). For N90 (artificial loci with a maximum of 181 genes, very close to the median size of 180 genes for OMIM phenotype loci with unknown molecular basis), for example, the leave-one-out validation yielded at least one candidate for 47.8% (Affy) and 12.7% (Stanford) of the disease loci (Figure 3A). In these cases, an average of 3.67 (Affy) and 2.17 (Stanford) candidates were returned (Figure 3B), that contained the true disease gene in 49.3% (Affy) and 43.6% (Stanford) of the cases (Figure 3C). That is, for both networks, when candidates were returned, the very short candidate lists contained the disease-causing gene with a probability of over 40%.

We next determined how our method performs compared to other existing approaches. This comparison was based on the enrichment in correctly identified disease genes (by leave-one-out) with respect to randomized networks. In our case the fold enrichment is defined as the ratio between number of disease genes correctly recalled in the leave-one out procedure and the same number averaged on 100 randomized CCNs. For this evaluation we used the N90 artificial loci (see Materials and Methods), which are closest in size to the actual orphan loci (median size 180). Fold enrichment values were computed for several published methods by Lage et al [8] (see their Supplementary material). It must be noticed that the exact definition of the fold enrichment depends on the method under consideration, thus comparison must be taken with caution. However, Table 1 shows that our methods compares favorably with many previously published ones: when considering that, as discussed above, the use of gene expression data allows a less biased analysis compared to most other methods, we conclude that our approach provides a significant and original contribution to the identification of disease genes.

**Table 1:** Fold enrichment of different methods for candidate gene prioritization.

Method	Data Source	Fold Enrichment
Lage et al. [8]	PPI	23.1^a^
Perez-Iratxeta et al. [35]	FA	19.4^a^
***Conserved coexpression (Affy network)***	GE	**14.4**
Freudenberg and Propping [36]	FA	13.3^a^
***Conserved coexpression (Stanford network)***	GE	**10.0**
Oti et al. [10]	PPI	10.0^a^
Adie et al. [37]	SBF	5.6^a^
Turner et al. [6]	FA	5.2^a^
Franke et al. [5]	FA, GE, PPI	3.6^a^

PPI, protein-protein interaction; FA, functional annotation; GE, gene expression; SBF, sequence-based features (e.g., sequence length).

a Values taken from the supplementary material of Lage et al. [8].

### Prediction of Candidates for Orphan Disease Loci

The above results indicate that conserved coexpression can be efficiently combined with phenotype correlation data to provide high confidence candidates within genetic disease loci. Therefore, we applied our procedure to 850 OMIM phenotype entries with at least one mapped disease locus but unknown molecular basis. In Table 2 we provide the list of all the 321 candidates (gene-locus pairs) obtained with 10% FDR. We obtained predictions for 81 loci, 67 of which where only from the Affy network, 5 only from the Stanford and 9 from both. Interestingly, in 4 of the latter cases, the list of candidates from the two networks contained at least one common gene (Table 2).

**Table 2:** List of the candidate genes found for 81 OMIM loci with unknown molecular basis.

OMIM ID	Disease and Locus	Locus Size	HUGO	Ensembl ID	Status	Net.	Disease Cluster Size	P-Value
119540	Cleft palate, isolated; 2q32	75	COL3A1	ENSG00000168542	1*(i)	S	22	1.16E-06
			COL5A2	ENSG00000204262	1*(i)	S	22	1.16E-06
121210	Febrile convulsions, familial, 1; 8q13-q21	188	C8orf46	ENSG00000169085	1	A	143	4.12E-10
			EFCBP1	ENSG00000123119	1	A	143	4.12E-10
			RALYL	ENSG00000184672	1	A	143	4.12E-10
			STMN2	ENSG00000104435	1	A	143	4.12E-10
			GDAP1	ENSG00000104381	1	A	139	1.79E-09
			C8orf34	ENSG00000165084	1	A	110	2.95E-08
			STAU2	ENSG00000040341	1	A	173	3.08^E^-08
130080	Ehlers-Danlos syndrome, type VIII; 12p13	277	A2M	ENSG00000175899	1	S	50	4.79E-11
			VWF	ENSG00000110799	1	A	99	9.97E-10
			C1S	ENSG00000182326	1	S	87	1.25E-09
			MFAP5	ENSG00000197614	1	A	111	1.90E-09
			EMP1	ENSG00000134531	1	A	72	2.37E-09
			CD163	ENSG00000177575	1	A	44	3.34E-09
			TNFRSF1A	ENSG00000067182	1	A	61	4.34E-09
			TSPAN9	ENSG00000011105	1	S	112	5.28E-09
			CSDA	ENSG00000060138	1	A	55	9.95E-09
			LTBR	ENSG00000111321	1	A	73	1.70E-06
			CD9	ENSG00000010278	1	S;A	49	2.34E-06
142700	Acetabular dysplasia; 13q22	36	KLF5	ENSG00000102554	1	A	54	1.18E-06
145410	Hypertelorism with esophageal abnormality and hypospadias; 22q11.2	271	KLHL22	ENSG00000185214	1	A	15	1.84E-07
			MAPK1	ENSG00000100030	1	A	15	1.84E-07
			MICAL3	ENSG00000099972	1	A	15	1.84E-07
154275	Malignant hyperthermia, susceptibility to, 2; 17q11.2-q24	881	CACNB1	ENSG00000067191	3$(ii)	A	37	1.06E-06
			CACNG1	ENSG00000108878	3$(ii)	A	37	1.06E-06
			SCN4A	ENSG00000007314	1	A	37	1.06E-06
156232	Mesomelic dysplasia, Kantaputra type; 2q24-q32	296	HOXD10	ENSG00000128710	2	A	13	1.80E-07
			HOXD8	ENSG00000175879	1	A	13	1.80E-07
			GRB14	ENSG00000115290	1	A	63	3.14E-07
			HOXD11	ENSG00000128713	1	A	63	3.14E-07
			HOXD3	ENSG00000128652	1	A	63	3.14E-07
156600	Microcoria, congenital; 13q31-q32	99	DCT	ENSG00000080166	1	A	18	4.06E-08
			SLITRK6	ENSG00000184564	1	A	18	4.06E-08
162820	Neutrophil chemotactic response; 7q22-qter	712	EPHB6	ENSG00000106123	1	A	46	6.04E-07
			EPHA1	ENSG00000146904	1	A	54	1.08E-06
163000	Nevi flammei, familial multiple; 5q13-q22	327	AGGF1	ENSG00000164252	2*(iii)	A	77	3.72E-08
			RASA1	ENSG00000145715	2#(iii)	A	77	3.72E-08
164210	Hemifacial microsomia; 14q32	414	BTBD7	ENSG00000011114	1	S	109	2.15E-07
			DICER1	ENSG00000100697	1	S	109	2.15E-07
			EIF5	ENSG00000100664	1	S	109	2.15E-07
			JAG2	ENSG00000184916	1	S	109	2.15E-07
			PAPOLA	ENSG00000090060	1	S	109	2.15E-07
			PPP2R5C	ENSG00000078304	1	S	109	2.15E-07
			VRK1	ENSG00000100749	1	S	109	2.15E-07
177720	Pseudohyperkalemia, familial, 1, due to red cell leak; 16q23-q24	170	CA5A	ENSG00000174990	1	A	11	1.56E-07
180020	Retinal cone dystrophy 1; 6q25-q26	133	PLEKHG1	ENSG00000120278	1	A	18	1.10E-09
			SYTL3	ENSG00000164674	1	A	18	1.10E-09
181430	Scapuloperoneal myopathy; 12q15-q23.1	215	MYF6	ENSG00000111046	3	A	104	8.24E-09
			PHLDA1	ENSG00000139289	1	A	134	3.50E-08
			LUM	ENSG00000139329	1	A	90	3.66E-07
183600	Split-hand/foot malformation 1; 2q31	126	HOXD10	ENSG00000128710	1	A	27	8.16E-10
			HOXD11	ENSG00000128713	1	A	27	8.16E-10
			HOXD3	ENSG00000128652	1	A	27	8.16E-10
			HOXD8	ENSG00000175879	1	A	27	8.16E-10
			HOXD9	ENSG00000128709	1	A	27	8.16E-10
			HOXD13	ENSG00000128714	2	A	28	2.67E-07
185000	Stomatocytosis I; 9q34.1	135	LCN2	ENSG00000148346	1	A	35	3.34E-07
203650	Alopecia-mental retardation syndrome 1; 3q26.3-q27.3	150	ABCC5	ENSG00000114770	1	S	23	6.51E-06
			LIPH	ENSG00000163898	2	S	23	6.51E-06
213200	Spinocerebellar ataxia, autosomal recessive 2; 9q34-qter	290	CACNA1B	ENSG00000148408	1	A	31	2.00E-06
			GRIN1	ENSG00000176884	1	A	50	3.38E-06
213600	Basal ganglia calcification, idiopathic, 1; 14q	1215	GPHN	ENSG00000171723	1	A	61	2.11E-06
214900	Cholestasis-lymphedema syndrome; 15q	1074	CYP1A2	ENSG00000140505	1	A	73	2.66E-06
			LIPC	ENSG00000166035	1	A	73	2.66E-06
218400	Craniometaphyseal dysplasia, autosomal recessive; 6q21-q22	215	GJA1	ENSG00000152661	1	S	65	8.67E-07
225000	Rosselli-gulienetti syndrome; 11q23-q24	314	BACE1	ENSG00000186318	1	S	65	4.88E-07
			CRYAB	ENSG00000109846	1	S	65	4.88E-07
			TAGLN	ENSG00000149591	1	S	65	4.88E-07
255160	Myopathy, hyaline body, autosomal recessive; 3p22.2-p21.32	84	CMYA1	ENSG00000168334	1	A	119	4.01E-18
			HHATL	ENSG00000010282	1	A	119	4.01E-18
			SCN5A	ENSG00000183873	1	A	83	1.74E-09
259450	Bruck syndrome 1; 17p12	37	MYOCD	ENSG00000141052	1	A	67	3.43E-06
			PMP22	ENSG00000109099	1	A	68	3.64E-06
268700	Saccharopinuria; 7q31.3	39	AASS	ENSG00000008311	2#(iv)	A	141	9.48E-15
			SLC13A1	ENSG00000081800	1*(iv)	A	88	1.80E-09
			TSPAN12	ENSG00000106025	1*(iv)	A	56	3.35E-07
300046	Mental retardation, X-linked 23; Xq23-q24	116	ACSL4	ENSG00000068366	2	A	36	2.38E-06
			WDR44	ENSG00000131725	1	A	36	2.38E-06
300148	Mental retardation, epileptic seizures, hypogonadism and hypogenitalism, microcephaly, and obesity; Xp22.13-p21.1	117	PDHA1	ENSG00000131828	1	A	40	1.03E-09
300195	Alport syndrome, mental retardation, midface hypoplasia, and elliptocytosis; Xq22.3	40	COL4A5	ENSG00000188153	2#(v)	A	20	3.85E-06
300324	Mental retardation, X-linked 53; Xq22.2-q26	327	ACSL4	ENSG00000068366	2	A	19	1.41E-06
			ARHGEF6	ENSG00000129675	2	A	19	1.41E-06
300489	Spinal muscular atrophy, distal, x-linked recessive; Xq13.1-q21	182	ITGB1BP2	ENSG00000147166	1	A	60	2.41E-06
			SH3BGRL	ENSG00000131171	1	A	26	3.16E-06
309610	Prieto X-linked mental retardation syndrome; Xp11-q21	481	ATRX	ENSG00000085224	2	S	24	6.25E-08
			HUWE1	ENSG00000086758	1	S	24	6.25E-08
			OGT	ENSG00000147162	1	S	24	6.25E-08
			USP9X	ENSG00000124486	1	S	24	6.25E-08
			MAGED1	ENSG00000179222	1	A	21	1.13E-07
			MAGED2	ENSG00000102316	1	A	21	1.13E-07
			DIAPH2	ENSG00000147202	1	S	17	2.28E-06
			CRSP2	ENSG00000180182	1	S	107	6.20E-06
			RNF12	ENSG00000131263	1	S	107	6.20E-06
			UTX	ENSG00000147050	1	S	107	6.20E-06
310440	Myopathy, X-linked, with excessive autophagy; Xq28	151	SLC6A8	ENSG00000130821	1	A	76	8.25E-11
			SRPK3	ENSG00000184343	1	A	62	1.12E-09
			IL9R	ENSG00000124334	1	A	79	6.05E-09
			DNASE1L1	ENSG00000013563	1	A	29	6.40E-08
			BGN	ENSG00000182492	1	S	112	7.32E-08
311510	Parkinsonism, early-onset, with mental retardation; Xq28	151	BCAP31	ENSG00000185825	1	S	26	3.48E-07
			IRAK1	ENSG00000184216	1	S	26	3.48E-07
			SSR4	ENSG00000180879	1	S	26	3.48E-07
			RAB39B	ENSG00000155961	1	A	66	2.02E-06
314580	Wieacker syndrome; Xq13-q21	182	ITGB1BP2	ENSG00000147166	1	A	87	5.02E-08
			PHKA1	ENSG00000067177	1	A	87	5.02E-08
			MAGEE1	ENSG00000198934	1	A	144	1.89E-07
			APOOL	ENSG00000155008	1	A	54	2.97E-06
600131	Epilepsy, childhood absence, 1; 8q24	247	NIBP	ENSG00000167632	1	A	139	1.38E-07
			LYNX1	ENSG00000180155	1	A	173	4.55E-07
			BAI1	ENSG00000181790	1	A	88	8.59E-07
600175	Spinal muscular atrophy, distal, congenital nonprogressive; 12q23-q24	445	HSPB8	ENSG00000152137	2	A	108	1.88E-07
			MYBPC1	ENSG00000196091	1	A	108	1.88E-07
			MYL2	ENSG00000111245	1	A	99	2.33E-06
600593	Craniosynostosis, Adelaide type; 4p16	160	MSX1	ENSG00000163132	1	A	28	6.29E-07
600624	Cone-rod dystrophy 1; 18q21.1-q21.3	148	SERPINB2	ENSG00000197632	1	A	63	3.75E-06
			SERPINB4	ENSG00000057149	1	A	63	3.75E-06
			SERPINB5	ENSG00000206075	1	A	63	3.75E-06
			SERPINB7	ENSG00000166396	1	A	63	3.75E-06
600792	Deafness, neurosensory, autosomal recessive 5; 14q12	43	COCH	ENSG00000100473	2	A	31	1.36E-06
			FOXG1B	ENSG00000176165	1	A	31	1.36E-06
600964	Refsum disease with increased pipecolic acidemia; 10pter-p11.2	312	PHYH	ENSG00000107537	2	A	46	3.04E-06
600977	Cone-rod dystrophy 5; 17p13-p12	319	RCVRN	ENSG00000109047	1*( vi)	A	12	1.04E-06
			PITPNM3	ENSG00000091622	1*( vi)	A	10	3.62E-06
601202	Cataract, anterior polar, 2; 17p13	282	RCVRN	ENSG00000109047	1	A	12	3.53E-07
601251	Retinal cone dystrophy 2; 17p	494	RCVRN	ENSG00000109047	1*(vii)	A	12	4.20E-07
			PITPNM3	ENSG00000091622	1*(vii)	A	10	2.65E-06
601362	Digeorge syndrome/velocardiofacial syndrome spectrum of malformation 2; 10p14-p13	71	GATA3	ENSG00000107485	2	A	29	1.75E-07
601676	Acute insulin response; 1p31	156	ANGPTL3	ENSG00000132855	1	A	46	4.21E-08
			CTH	ENSG00000116761	1	A	46	4.21E-08
			CRYZ	ENSG00000116791	1	A	37	1.84E-07
601764	Convulsions, benign familial infantile, 1; 19q	1002	ATP1A3	ENSG00000105409	1	A	100	2.71E-08
			FXYD7	ENSG00000142290	1	A	100	2.71E-08
			SCN1B	ENSG00000105711	2$(viii)	A	100	2.71E-08
			APLP1	ENSG00000105290	1	A	82	6.25E-08
			CA11	ENSG00000063180	1	A	82	6.25E-08
			TTYH1	ENSG00000167614	1	A	82	6.25E-08
			LRRC4B	ENSG00000131409	1	A	125	4.11E-07
			MAG	ENSG00000105695	1	A	125	4.11E-07
			TMEM145	ENSG00000167619	1	A	125	4.11E-07
			ZNF536	ENSG00000198597	1	A	125	4.11E-07
			ZNF8	ENSG00000083842	1	A	173	7.17E-07
			CPT1C	ENSG00000169169	1	A	115	8.94E-07
			CADM4	ENSG00000105767	1	A	73	9.65E-07
			LIN7B	ENSG00000104863	1	A	139	1.05E-06
			PLD3	ENSG00000105223	1	A	139	1.05E-06
			SPTBN4	ENSG00000160460	1	A	139	1.05E-06
			GRIK5	ENSG00000105737	1	A	88	3.73E-06
601846	Vacuolar neuromyopathy; 19p13.3	238	ITGB1BP3	ENSG00000077009	1	A	119	2.44E-14
			NRTN	ENSG00000171119	1	A	108	2.89E-13
			GNG7	ENSG00000176533	1	A	144	2.41E-10
			PRTN3	ENSG00000196415	1	A	113	1.20E-09
			MKNK2	ENSG00000099875	1	A	45	2.27E-07
			TRIP10	ENSG00000125733	1	A	45	2.27E-07
602067	Cardiomyopathy, dilated, 1f; 6q23	74	AHI1	ENSG00000135541	1	A	144	3.32E-14
			EYA4	ENSG00000112319	1	A	134	9.27E-13
			TCF21	ENSG00000118526	1	A	33	3.69E-12
			HEBP2	ENSG00000051620	1	A	72	2.44E-06
603165	Dermatitis, atopic; 1q21	334	ANXA9	ENSG00000143412	1	A	72	2.24E-09
			ECM1	ENSG00000143369	1	A	72	2.24E-09
			FLG	ENSG00000143631	2	A	72	2.24E-09
			LOR	ENSG00000203782	1	A	72	2.24E-09
			S100A14	ENSG00000189334	1	A	72	2.24E-09
			SPRR1B	ENSG00000169469	1	A	72	2.24E-09
603204	Epilepsy, nocturnal frontal lobe, type 2; 15q24	97	LINGO1	ENSG00000169783	1	A	68	2.45E-08
			SCAMP5	ENSG00000198794	1	A	51	9.65E-08
603511	Muscular dystrophy, limb-girdle, type 1d; 7q	1069	ASB10	ENSG00000146926	1	A	72	8.28E-09
			ASB15	ENSG00000146809	1	A	72	8.28E-09
			FLNC	ENSG00000128591	3(ix)	A	72	8.28E-09
			PPP1R3A	ENSG00000154415	1	A	72	8.28E-09
			PDK4	ENSG00000004799	1	A	120	8.73E-09
			MYLC2PL	ENSG00000106436	1	A	119	9.20E-08
			CD36	ENSG00000135218	1	A	144	1.13E-07
			EXOC4	ENSG00000131558	1	A	144	1.13E-07
			EPHA1	ENSG00000146904	1	A	26	2.89E-07
			FOXP2	ENSG00000128573	1	A	26	2.89E-07
603786	Stargardt disease 4; 4p	361	APBB2	ENSG00000163697	1	S	77	2.34E-06
			FAM114A1	ENSG00000197712	1	S	77	2.34E-06
			UGDH	ENSG00000109814	1	S	77	2.34E-06
604288	Cardiomyopathy, dilated, 1h; 2q14-q22	278	ACVR2A	ENSG00000121989	1	A	134	2.24E-08
			HNMT	ENSG00000150540	1	A	134	2.24E-08
604364	Epilepsy, partial, with variable foci; 22q11-q12	474	CACNG2	ENSG00000166862	1	A	73	1.18E-07
			SEZ6L	ENSG00000100095	1	A	73	1.18E-07
			SLC25A18	ENSG00000182902	1	A	73	1.18E-07
			GANZ	ENSG00000128266	1	A	51	8.91E-07
			C22orf25	ENSG00000183597	1	A	39	1.31E-06
604454	Welander distal myopathy; wdm; 2p13	111	HK2	ENSG00000159399	1	A	107	1.01E-13
			ANTXR1	ENSG00000169604	1	S;A	98	1.75E-06
			ANXA4	ENSG00000196975	1	A	73	3.04E-06
604499	Hyperlipidemia, combined, 2; 11p	637	F2	ENSG00000180210	1	A	103	1.02E-06
			HPX	ENSG00000110169	1	A	103	1.02E-06
			SAA4	ENSG00000148965	1	A	103	1.02E-06
604781	Ichthyosis, nonlamellar and nonerythrodermic, congenital, autosomal recessive; 19p13.2-p13.1	434	ABHD9	ENSG00000105131	1	A	57	4.18E-14
			CASP14	ENSG00000105141	1	A	57	4.18E-14
			CYP4F22	ENSG00000171954	1	A	57	4.18E-14
			KIAA1543	ENSG00000076826	1	A	33	5.44E-10
604801	Muscular dystrophy, congenital, 1b; 1q42	194	ACTA1	ENSG00000143632	2	A	119	1.66E-16
			CABC1	ENSG00000163050	1	A	119	1.66E-16
			NID1	ENSG00000116962	1	S	112	4.04E-08
			C1orf198	ENSG00000119280	1	S	68	6.50E-07
			ABCB10	ENSG00000135776	1	S	98	6.70E-07
605021	Myoclonic epilepsy, infantile; 16p13	366	A2BP1	ENSG00000078328	1	A	100	3.94E-08
			CASKIN1	ENSG00000167971	1	A	100	3.94E-08
			MAPK8IP3	ENSG00000138834	1	A	100	3.94E-08
			SYNGR3	ENSG00000127561	1	A	139	8.45E-07
			FLYWCH1	ENSG00000059122	1	A	173	1.72E-06
605285	Neuropathy, hereditary motor and sensory, russe type; 10q23.2	26	SNCG	ENSG00000173267	1	A	32	5.27E-09
605480	Systemic lupus erythematosus, susceptibility to; 4p16-p15.2	239	CRMP1	ENSG00000072832	1	A	51	1.21E-06
			KCNIP4	ENSG00000185774	1	A	51	1.21E-06
			LGI2	ENSG00000153012	1	A	51	1.21E-06
			NSG1	ENSG00000168824	1	A	52	1.32E-06
			PPP2R2C	ENSG00000074211	1	A	52	1.32E-06
605582	Cardiomyopathy, dilated, 1k; 6q12-q16	209	ME1	ENSG00000065833	3$(x)	A	76	9.60E-08
			TPBG	ENSG00000146242	1	A	134	4.83E-07
605642	Thyroid carcinoma, papillary, with papillary renal neoplasia; 1q21	334	MRPL9	ENSG00000143436	1	A	9	1.17E-06
			PRKAB2	ENSG00000131791	1	A	58	2.97E-06
605711	Multiple mitochondrial dysfunctions syndrome; 2p14-p13	142	MCEE	ENSG00000124370	1	A	54	1.28E-06
605751	Convulsions, benign familial infantile, 2; 16p12-q12	407	GNAO1	ENSG00000087258	1	A	173	4.17E-08
			MT3	ENSG00000087250	1	A	173	4.17E-08
			SEZ6L2	ENSG00000174938	1	A	84	2.67E-07
			ITFG1	ENSG00000129636	1	A	159	1.14E-06
			USP31	ENSG00000103404	1	A	159	1.14E-06
			CE110	ENSG00000103540	1	A	157	2.84E-06
			PRKCB1	ENSG00000166501	1	A	157	2.84E-06
605809	Myasthenia, familial infantile, 1; 17p13	282	hCG_1776018	ENSG00000188265	1	A	32	1.86E-06
			CHRNB1	ENSG00000170175	2£(xi)	A	68	3.01E-06
			ENO3	ENSG00000108515	1	A	68	3.01E-06
			MYH1	ENSG00000109061	1	A	68	3.01E-06
606070	Myopathy, distal 2; 5q	1116	MYOT	ENSG00000120729	2	A	120	2.33E-09
			THBS4	ENSG00000113296	1	A	120	2.33E-09
			DBN1	ENSG00000113758	1	S	45	3.10E-08
			LOX	ENSG00000113083	1	S	45	3.10E-08
			LOC493869	ENSG00000164294	1	S	45	3.10E-08
			SLIT3	ENSG00000184347	1	S	45	3.10E-08
			SPARC	ENSG00000113140	1	S	45	3.10E-08
			HSPB3	ENSG00000169271	1	A	114	5.19E-08
			MEF2C	ENSG00000081189	1	A	114	5.19E-08
			AFAP1L1	ENSG00000157510	1	A	144	6.16E-08
			EDIL3	ENSG00000164176	1	A	144	6.16E-08
			FGF1	ENSG00000113578	1	A	144	6.16E-08
			GABRG2	ENSG00000113327	1	A	144	6.16E-08
			SCAMP1	ENSG00000085365	1	A	144	6.16E-08
			CTNNA1	ENSG00000044115	1	S	84	7.06E-08
			EGR1	ENSG00000120738	1	S	84	7.06E-08
			IL6ST	ENSG00000134352	1	S	84	7.06E-08
			NR2F1	ENSG00000175745	1	S	84	7.06E-08
			PCDHGA12	ENSG00000081853	1	S	84	7.06E-08
			PPIC	ENSG00000168938	1	S	84	7.06E-08
			VCAN	ENSG00000038427	1	S	84	7.06E-08
			GABRP	ENSG00000094755	1	A	62	9.02E-08
			SPINK5	ENSG00000133710	1	A	120	9.28E-08
			SYNPO	ENSG00000171992	1	S	65	4.99E-07
			EFNA5	ENSG00000184349	1	S	75	5.90E-07
			PDGFRB	ENSG00000113721	1	S	75	5.90E-07
			PAM	ENSG00000145730	1	S	112	1.27E-06
			REEP5	ENSG00000129625	1	S	112	1.27E-06
			LAEVR	ENSG00000172901	1	A	44	1.35E-06
			MAP1B	ENSG00000131711	1	S	98	2.10E-06
			P4HA2	ENSG00000072682	1	S	98	2.10E-06
			DPYSL3	ENSG00000113657	1	S	90	2.34E-06
			NR3C1	ENSG00000113580	1	S	64	2.39E-06
			GFM2	ENSG00000164347	1	A	68	2.53E-06
			U384	ENSG00000048162	1	A	68	2.53E-06
			CKMT2	ENSG00000131730	1	A	68	3.90E-06
606257	Stature quantitative trait locus 3; 12p11.2-q14	548	KRT1	ENSG00000167768	1	A	72	1.32E-06
			KRT2	ENSG00000172867	1	A	72	1.32E-06
			KRT71	ENSG00000139648	1	A	72	1.32E-06
			KRT8	ENSG00000170421	1	A	72	1.32E-06
			PP11	ENSG00000111405	1	A	72	1.32E-06
606483	Charcot-Marie-Tooth disease, dominant intermediate A; 10q24.1-q25.1	179	SFRP5	ENSG00000120057	1	A	69	1.61E-06
606545	Ichthyosis, lamellar, 5; 17p13.2-p13.1	210	ALOX12B	ENSG00000179477	2	A	58	1.78E-17
			ALOXE3	ENSG00000179148	2	A	58	1.78E-17
			GGT6	ENSG00000167741	1	A	58	1.78E-17
			ENO3	ENSG00000108515	1	A	71	1.65E-16
			SOX15	ENSG00000129194	1	A	41	3.05E-10
			MYH2	ENSG00000125414	1	A	65	1.35E-09
			CLDN7	ENSG00000181885	1	A	186	5.49E-07
606708	Split-hand/foot malformation 5; 2q31	126	HOXD10	ENSG00000128710	1	A	13	4.55E-07
			HOXD8	ENSG00000175879	1	A	13	4.55E-07
			HOXD11	ENSG00000128713	1	A	63	1.67E-06
			HOXD3	ENSG00000128652	1	A	63	1.67E-06
			HOXD13	ENSG00000128714	2$(xii)	A	28	2.08E-06
606744	Seckel syndrome 2; 18p11.31-q11.2	169	VAPA	ENSG00000101558	1	A	36	3.68E-06
607086	Aortic aneurysm, familial thoracic 1; 11q23.3-q24	250	TAGLN	ENSG00000149591	1	A	92	8.74E-07
			MCAM	ENSG00000076706	1	A	138	1.83E-06
607088	Spinal muscular atrophy, distal, autosomal recessive, 3; 11q13	354	ACTN3	ENSG00000204633	1	A	108	2.28E-10
			PYGM	ENSG00000068976	1	A	108	2.28E-10
			EHBP1L1	ENSG00000173442	1	A	87	5.07E-08
			LRP16	ENSG00000133315	1	A	144	5.11E-08
			P2RY2	ENSG00000175591	1	A	120	1.87E-07
			CCND1	ENSG00000110092	1	S	98	3.99E-07
			SERPINH1	ENSG00000149257	1	S;A	98	3.99E-07
			PLCB3	ENSG00000149782	1	A	52	1.03E-06
			PDE2A	ENSG00000186642	1	S	65	1.72E-06
			FOLR2	ENSG00000165457	1	A	85	1.82E-06
			UCP3	ENSG00000175564	1	A	85	1.82E-06
			CTTN	ENSG00000085733	1	S	79	1.84E-06
			CD248	ENSG00000174807	1	A	72	2.05E-06
			DGAT2	ENSG00000062282	1	A	75	2.55E-06
			ARHGEF17	ENSG00000110237	1	S	84	2.68E-06
607221	Epilepsy, partial, with pericentral spikes; 4p15	86	KCNIP4	ENSG00000185774	1	A	143	1.65E-07
			LGI2	ENSG00000153012	1	A	51	2.31E-07
607936	Exfoliative ichthyosis, autosomal recessive, ichthyosis bullosa of siemens-like; 12q13	355	KRT1	ENSG00000167768	2	A	57	6.70E-20
			KRT2	ENSG00000172867	2	A	57	6.70E-20
			KRT8	ENSG00000170421	1	A	57	6.70E-20
			KRT84	ENSG00000161849	1	A	57	6.70E-20
			SDR-O	ENSG00000170426	1	A	57	6.70E-20
			KRT71	ENSG00000139648	1	A	52	1.55E-19
			PP11	ENSG00000111405	1	A	61	1.06E-18
			VDR	ENSG00000111424	1	A	61	1.06E-18
			KRT4	ENSG00000170477	1	A	71	1.97E-15
			KRT76	ENSG00000185069	1	A	67	2.51E-13
			RARG	ENSG00000172819	1	A	76	2.38E-11
			GLS2	ENSG00000135423	1	A	33	4.03E-09
			SLC38A4	ENSG00000139209	1	A	33	4.03E-09
			HOXC13	ENSG00000123364	1	A	117	4.15E-09
			KRT82	ENSG00000161850	1	A	41	8.03E-07
608096	Epilepsy, familial temporal lobe; 12q22-q23.3	163	ANKS1B	ENSG00000185046	1	A	143	1.04E-06
608224	Deafness, autosomal dominant nonsyndromic sensorineural 41; 12q24.32-qter	71	POLE	ENSG00000177084	1	A	19	1.00E-06
			ULK1	ENSG00000177169	1	A	19	1.00E-06
608318	Coronary heart disease, susceptibility to, 4; 14q32	414	SERPINA10	ENSG00000140093	1	A	9	1.25E-06
608423	Muscular dystrophy, limb-girdle, type 1f; 7q32.1-q32.2	71	FAM40B	ENSG00000128578	1	A	113	4.50E-11
			FLNC	ENSG00000128591	2(ix)	S;A	112	3.44E-08
			LEP	ENSG00000174697	1	A	24	1.45E-06
608762	Epilepsy, idiopathic generalized, susceptibility to, 3; 9q32-q33	161	GOLGA1	ENSG00000136935	1	A	173	4.49E-06
608816	Myoclonic epilepsy, juvenile, 3; 6p21	395	PACSIN1	ENSG00000124507	1	A	173	1.01E-07

The column ‘status’ reviews the current knowledge about the association of the candidate with the disease. In particular, 1 = gene not previously associated with the disease; 2 = gene involved in mendelian phenotype sharing a MimMiner similarity score of 0.4 or higher with the phenotypic description of the locus; 3 = gene previously considered as a candidate for clinical similarity, but with a MimMiner similarity score to the locus lower than 0.4. Moreover, genes annotated with “#” represent the actual disease gene, because mutations have been found in patients; genes annotated with “£” have been excluded by refining the map interval; genes annotated with “*” could be excluded because mutations have been found in a different gene of the same locus; genes annotated with “$” are at the moment excluded because they have been screened but no mutations considered to be relevant have been found. The above statements are supported by the indicated references: i = [38]; ii = [39]; iii = [40]; iv = [41]; v = [42]; vi = [43]; vii = [44]; viii = [45]; ix = [46]; x = [47]; xi = [48]; xii = [49]. The column “Net.” indicates the networks from which the candidate was predicted: A = Affy; S = Stanford.

Notably, for three OMIM phenotypes (163000, familial multiple nevi flammei; 268700, saccharopinuria; 300195; AMMECR1) our predictions include the actual disease genes that, although not yet correctly annotated in OMIM, have been found to be mutated in patients (see Table 2).

For 22 loci, at least one of the candidates obtained from either network was already known to be involved in phenotypes similar to those described for the locus. These genes represent the most obvious candidates and our results should be considered as further, independent evidence for their possible involvement in the disease. However, it must be noted that some of them were previously excluded, either by the direct identification of crossovers or by the negative results of mutation screenings. Nevertheless, since mutations have most likely been searched only within the annotated exons, we think that the decision to definitively rule out the involvement of such candidates should be taken cautiously. Moreover, even silent exonic mutations, although often considered innocuous polymorphisms, can have severe effects on proteins by disrupting splicing patterns [27],[28].

In most cases only few candidates are given for a locus, thus providing extremely focused working hypotheses for the identification of the actual disease genes, which in many cases are made even stronger by the available sequence or functional information.

**Figure 4:**
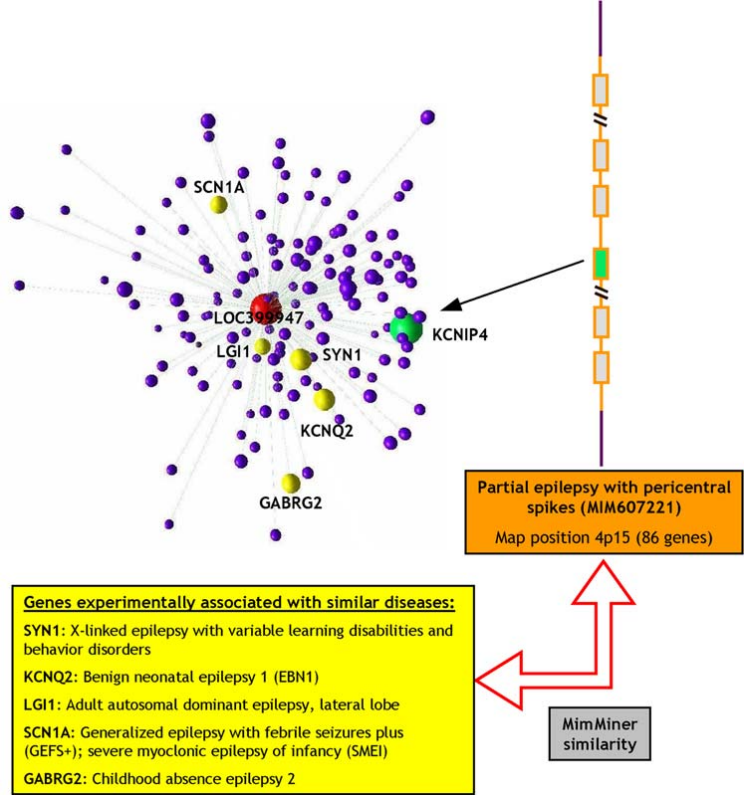
Example of identification of candidate disease genes. Identification of KCNIP4 (green) in the genomic locus 4p15 (containing 86 genes; orange), associated with partial epilepsy with pericentral spikes (OMIM ID 607221). KCNIP4 was found in a disease-related cluster composed of LOC399947 and its nearest neighbors (red and purple spheres, respectively) that contains 5 other genes known to be involved in similar phenotypes (yellow spheres). A second candidate for this disease was found in another disease-associated cluster (not shown; see Table 2).

For instance, one of the two candidates provided for the OMIM phenotype entry 607221 (partial epilepsy with pericentral spikes, located on 4p15) corresponds to KCNIP4 (Figure 4). This protein has been show to specifically modulate the activity of Kv4 A-type potassium channels [29], which are well known regulators of membrane excitability [30] and have been recently involved in epilepsy [31]. Another interesting example is given by the 605285 phenotype entry (hereditary motor and sensory neuropathy, Russe type, mapped to 10q23.2, a locus comprising only 26 candidate genes). The only prediction for this locus is gamma-synuclein (SNCG), which is a very strong candidate both for the low P-value and for the known role of synucleins in neurodegenerative disorders [32].

Even when the number of candidates for a particular locus is substantially higher, our results may provide a strong restriction of the experimental search field, which can be further narrowed by additional evidences. For instance, the phenotype with OMIM ID 130080 (Ehlers-Danlos syndrome, type VIII), is mapped to 12p13, containing 277 genes. In this case, the Affy and Stanford networks provide 8 and 4 candidates, respectively. Interestingly, the candidate with the lowest associated P-value is the Alpha-2-macroglobulin precursor (A2M), whose absence was previously reported in a patient with Ehlers-Danlos syndrome [33]. A second interesting protein for this locus is CD9 that is the only candidate provided by both networks and that is known to regulate collagen matrix organization by interacting with Beta1 integrin [34].

In general, given the highly stringent criteria that we adopted and considering that the starting data underlying the two networks are completely independent, we propose that the 4 common candidates (Table 2) should be considered as those having the highest priority for experimental validation.

Since a recent study has identified high confidence candidate genes by integrating protein-protein interaction with phenotypic information [8], we evaluated the number of common predictions, and found that a candidate is proposed by both approaches for 7 loci. In 5 cases the candidate proposed by Lage et al. did not overlap with our predictions. The only, remarkable exception was Filamin C (FLNC), which was found as a candidate on chromosome 7q for both the 608423 (limb-girdle muscular dystrophy type 1F) and the 603511 (limb-girdle muscular dystrophy type 1D) OMIM phenotype entries. Interestingly, the FLNC gene has been previously implicated in myopathy, but mutations have not been reported in families mapping to the above loci. Thus, our results can be considered as further supporting evidence, pointing to the actual involvement of this gene in limb-girdle muscular dystrophy.

## Discussion

In the present report we have shown that the integration of massive gene expression data with phenotype similarity maps can allow to efficiently identify high-probability candidates for many orphan human genetic disease loci, even when these comprise hundreds of genes.

The comparison of the two different networks clearly showed that the Affy network is characterized by a much better signal/noise ratio than the Stanford network. Although this may be partially due to the different source material (normal tissues in the first case, mostly tumor cell lines in the second case) we think that this could also depend on the higher technical standards reached by oligonucleotide-based platforms. Nevertheless, it is also important to notice that the analysis of the Stanford network allowed the prediction of many candidates that were not obtained from the Affy network, indicating that different datasets can result in complementary predictions.

Interestingly, in both cases the gene coexpression criterion proved to be much more effective than it could be expected from previous work, where it has been mostly used in combination with other high-throughput information sources. We think that these results strongly underscore two critical points. The first is the importance of using a restrictive filter to select biologically relevant coexpression links, such as our phylogenetic filter selecting links which are under selective pressure and therefore more likely to imply functional relationships. The second is the usefulness of systematic phenotype analysis methods, which may capture disease similarities that could easily escape human operator-based approaches.

Although very significant under the statistical point of view, the overlapping between conserved-coexpression links and physical protein-protein interaction data appeared to be rather limited in absolute terms, strengthening the idea that these criteria may cover partially overlapping subsets of the functional interaction space. Our results strongly suggest that, in most of the cases, requiring the concordance of coexpression data and protein-protein interaction data may worsen, instead of improving, the performances of both methods. Therefore, we envisage the independent use of both types of evidence to predict functional relationships and candidate disease genes. However, in the limited cases for which these approaches provide convergent results, they can be used as strong additive evidence.

In conclusion, we propose that our method and our list of candidates will provide a useful support for the identification of new disease-relevant genes.

## Supporting Information

Text S1 Stanford human-mouse conserved coexpression network. Each row contains a link between two Entrez-Gene identifiers.
(0.66 MB TXT)

Text S2 Affy human-mouse conserved coexpression network. Each row contains a link between two Entrez-Gene identifiers.
(1.89 MB TXT)
